# Respiratory mechanics in infants with severe bronchiolitis on controlled mechanical ventilation

**DOI:** 10.1186/s12890-017-0475-6

**Published:** 2017-10-06

**Authors:** Pablo Cruces, Sebastián González-Dambrauskas, Julio Quilodrán, Jorge Valenzuela, Javier Martínez, Natalia Rivero, Pablo Arias, Franco Díaz

**Affiliations:** 1 Pediatric Intensive Care Unit, Hospital El Carmen de Maipú, Santiago, Chile; 2 Centro de Investigación de Medicina Veterinaria, Escuela de Medicina Veterinaria, Facultad de Ecología y Recursos Naturales, Universidad Andres Bello, Santiago, Chile; 3 Pediatric Intensive Care Unit, Centro Hospitalario Pereira Rossell, Montevideo, Uruguay; 4 Pediatric Intensive Care Unit, Clínica Alemana de Santiago, Vitacura, 5951 Santiago, Chile; 5 Facultad de Medicina Clínica Alemana Universidad del Desarrollo, Santiago, Chile

**Keywords:** Bronchiolitis, Mechanical ventilation, Respiratory mechanics, Work of breathing, Pediatrics

## Abstract

**Background:**

Analysis of respiratory mechanics during mechanical ventilation (MV) is able to estimate resistive, elastic and inertial components of the working pressure of the respiratory system. Our aim was to discriminate the components of the working pressure of the respiratory system in infants on MV with severe bronchiolitis admitted to two PICU’s.

**Methods:**

Infants younger than 1 year old with acute respiratory failure caused by severe bronchiolitis underwent neuromuscular blockade, tracheal intubation and volume controlled MV. Shortly after intubation studies of pulmonary mechanics were performed using inspiratory and expiratory breath hold. The maximum inspiratory and expiratory flow (QI and QE) as well as peak inspiratory (PIP), plateau (PPL) and total expiratory pressures (tPEEP) were measured. Inspiratory and expiratory resistances (RawI and RawE) and Time Constants (K_TI_ and K_TE_) were calculated.

**Results:**

We included 16 patients, of median age 2.5 (1–5.8) months. Bronchiolitis due to respiratory syncytial virus was the main etiology (93.8%) and 31.3% had comorbidities. Measured respiratory pressures were PIP 29 (26–31), PPL 24 (20–26), tPEEP 9 [8–11] cmH2O. Elastic component of the working pressure was significantly higher than resistive and both higher than threshold (tPEEP – PEEP) (*P* < 0.01). QI was significantly lower than QE [5 (4.27–6.75) v/s 16.5 (12–23.8) L/min. RawI and RawE were 38.8 (32–53) and 40.5 (22–55) cmH2O/L/s; K_TI_ and K_TE_ [0.18 (0.12–0.30) v/s 0.18 (0.13–0.22) s], and K_TI_:K_TE_ ratio was 1:1.04 (1:0.59–1.42).

**Conclusions:**

Analysis of respiratory mechanics of infants with severe bronchiolitis receiving MV shows that the elastic component of the working pressure of the respiratory system is the most important. The elastic and resistive components in conjunction with flow profile are characteristic of restrictive diseases. A better understanding of lung mechanics in this group of patients may lead to change the traditional ventilatory approach to severe bronchiolitis.

**Electronic supplementary material:**

The online version of this article (10.1186/s12890-017-0475-6) contains supplementary material, which is available to authorized users.

## Background

Respiratory infections are the leading cause of childhood mortality and morbidity worldwide. Bronchiolitis, the most common lower respiratory infection in infants, continues to be a major pediatric public health problem [1, 2]. Research over the past 30 years has led to significant improvement in our understanding of its pathophysiology, in identifying high-risk populations, and in attenuating the severity of the disorder [3–6].

Bronchiolitis is usually a self–limited disease, but some children may develop respiratory failure with increased work of breathing (WOB), hypoxemia and hypercarbia requiring mechanical ventilation (MV) in addition to usual supportive measures [7, 8]. Research has mostly focused on gas exchange and criteria for respiratory failure, and the efficacy of various treatments to improve gas exchange, reduce WOB, and hasten recovery [9]. Surprisingly, little work has been done studying respiratory mechanics in children with severe bronchiolitis under MV [4, 5].

Modern ventilators are thought to be not only a supportive machine, but also a bedside monitoring tool. Respiratory mechanics are the expression of lung function trough measures of pressure and flow. When positive pressure is applied to the respiratory system, advance analysis of respiratory mechanics can discriminate the different elements of WOB according to equation of motion: resistive component (the resistance to displacement of a determined gas flow), elastic component (elastic opposition to a determined change of volume) and a threshold load. The threshold load refers to the amount of work required to commence inspiratory flow and it is determined by autoPEEP [10–12]. Interpretation of these data can help to tailor MV parameters to match the pathophysiology of the disease according to which component is predominantly compromised. For example, contemporary MV strategies for severe asthma totally differ from acute respiratory distress syndrome (ARDS).

Bronchiolitis is usually considered an airway obstructive disease based on physical exam, although bronchodilator therapy has been proven to be ineffective. Groundbreaking studies 50 years ago described increased respiratory rate and decreased lung compliance in spontaneously breathing children with bronchiolitis [13]. A better characterization of respiratory mechanics in severe bronchiolitis is crucial to understand this disease to improve current ventilatory strategies and ultimately, it may improve usual PICU outcomes; like MV duration, respiratory support requirements, PICU and hospital length of stay.

With these facts in mind, we designed this study to examine the analysis of respiratory mechanics in infants on MV due to severe bronchiolitis. Our aim was to describe the work of breathing in this group of patients, analyzing the different components of the respiratory mechanics: the resistive and elastic forces, as well as threshold.

## Methods

### Study design and setting

This prospective observational study was conducted in 2 PICU’s: Centro Hospitalario Pereira Rossell is a 20 bed mixed medical surgical pediatric intensive care unit located in Montevideo, Uruguay, that covers all PICU pathologies except cardiosurgery; Hospital El Carmen de Maipú is a 6 bed polyvalent unit and a referral center for acute respiratory failure in Santiago, Chile.

### Study population

Between May 1st and August 28th, 2015, children younger than 1 year old with clinical diagnosis of bronchiolitis requiring MV due to acute respiratory failure were screened for the study. Patients were excluded if they had uncorrected congenital heart, pre-existing lung or airway disease, chronic respiratory failure requiring long–term MV and tracheostomy. Additionally, patients with spontaneous breathing effort, endotracheal tube air leak >20% of tidal volume (V_T_), consolidation or atelectasis greater than 2 quadrants on Antero-Posterior chest x-ray, bronchodilator administration 1 h before measurement at the time of measurement were excluded due to possible interference with data acquisition.

### Data collection

We registered demographics at admission, clinical information, Pediatric Index of Mortality 2 (PIM 2) and outcome. Patients were ventilated on Volume Control mode. Ventilator parameters [peak inspiratory pressure (PIP), plateau pressure (P_PL_), extrinsic PEEP (PEEP), total PEEP (tPEEP), driving pressure (∆P = P_PL_–tPEEP), expiratory V_T_ (VTE), inspiratory time (IT), and respiratory rate (RR)], maximum inspiratory and expiratory flow (QI and QE), and arterial blood gases before the measurement were registered and P_a_O_2_/F_i_O_2_ (PF) ratio and Oxygenation Index (OI) were calculated.

### Respiratory mechanics measurements and determination of components of working pressure

Each respiratory mechanics measurement was performed within 1 h after intubation by one of the investigators. Patients were sedated and under the effect of neuromuscular blocker with no respiratory effort. In this setting, we estimated the components of the working pressure of the respiratory system. In absence of respiratory muscle activity, working pressure of the respiratory system is the pressure needed to overcome frictional forces, elastic forces and impedance and it can be calculated applying the equation of motion:

$$ {\mathrm{P}}_{\mathrm{aw}}={\mathrm{V}}_{\mathrm{T}}/{\mathrm{C}}_{\mathrm{RS}}+{\mathrm{Raw}}_{\mathrm{I}}\ast {\mathrm{Q}}_{\mathrm{I}}+\mathrm{autoPEEP}. $$

**Fig. 1:**
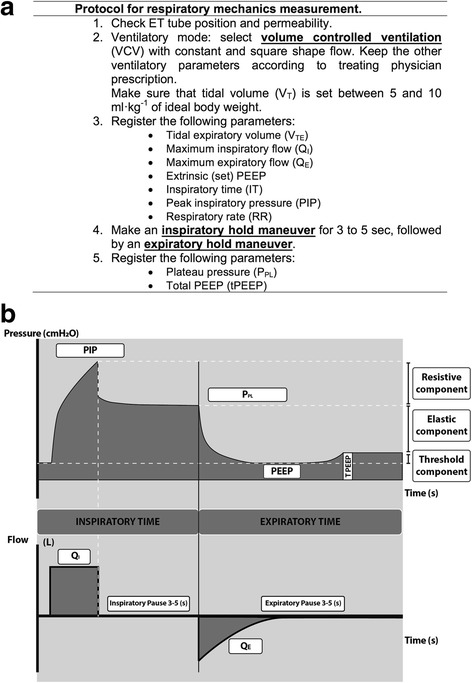
Respiratory mechanics measurement. **a** Respiratory mechanics measurement protocol. **b** Illustration of Airway Pressure versus time and flow versus time curves during inspiratory and expiratory breathhold. The components of work of breathing, elastic and threshold are represented

An inspiratory hold followed by an expiratory hold was performed following the protocol described in Fig. 1a. Flow and pressure parameters in these quasi static conditions at the Y piece (*proximal flow sensor*) were recorded in a ad–hoc Microsoft Excel 2010 (Microsoft®, NY, USA) database to calculate: respiratory system compliance (CRS, mL·cmH2O^−1^·kg^−1^), inspiratory and expiratory airway resistance (RawI and RawE, cmH2O·L^−1^·s^−1^), inspiratory and expiratory time constants (K_TI_ and K_TE_, s) according to formulas described in (Additional file 1: Table S1).

For each subject the components of working pressure, resistive, elastic and threshold were calculated and expressed as percentage of total working pressure of the respiratory system. Figure 1b shows an illustration of different components of working pressure and how they are measured on the mechanical ventilator. Each component of working pressure was expressed in cmH_2_O and as percentage of total working pressure of the respiratory system. Calculations were performed as follows: Resistive component: PIP – P_PL_; Elastic Component: P_PL_ – tPEEP; Threshold tPEEP – PEEP (or autoPEEP).

Mechanical ventilators used for measurements were Engstrom® (GE Datex, Madison, Wisconsin USA) and Hamilton G5 or Galileo® (Hamilton, Bonaduz, Switzerland), depending on the availability.

#### Data analysis

Data are expressed as means ± standard deviation (SD) and continuous data were expressed as median and interquartile range (IQR). 95% Confidence interval (CI95%) was calculated for proportions. Normality was assessed with the Anderson–Darling test. Kruskal–Wallis test with post hoc Dunns analysis was used for comparison of the working pressure components. T Student and Mann–Whitney tests for comparison of flow, resistance and time constants were used. Relation between variables was determined by Pearson correlation test. Significance was set at *P* < 0.05. All statistical analyses were performed using SPSS 20.0 (SPSS Inc., Chicago, IL, USA). Figures were plotted with GraphPad PRISM version 5.0c for Mac (GraphPad Software, La Jolla, CA, USA).

## Results

During the study period, 54 infants with bronchiolitis were screened. Thirty-one subjects were not enrolled due to age older than 1 year old, 5 excluded due to time on MV greater than 24 h at the time of measurements, 1 excluded due to non- corrected congenital heart disease and 1 excluded due to chronic lung disease. Sixteen patients were finally included in the study. Fifty percent were male, median age was 2.5 months (1–5.8), weight 5.9 kg (4.5–7.4).

Respiratory Syncytial Virus (RSV) was the main etiology, identified in 15 cases. Prematurity was main comorbidity, present in 5 patients. PIM2 Score was 7.5% (3.8–10.1), PF ratio was 195 mmHg (159–241) and OI was 7.1 (5.4–9.0). Table 1 shows clinical and gas exchange characteristics of included patients. Duration of MV was 5 days (IQR 2–7.25) and there was no mortality in this group.

**Table 1:** Clinical characteristics of patients with acute respiratory failure secondary to bronchiolitis included in the study

Patient	Age (mo)	Etiology	Comorbidities (1: No = 2: Yes)	PF ratio	PaCO_2_	OI	PIM2 (%)	Outcome
1	1	RSV	1	280	42	5.6	10.1	Alive
2	1	RSV	1	293	37	4.2	10.1	Alive
3	1	RSV	1	127	112	10.3	5.1	Alive
4	1	RSV	2: PNB 36 week	224	65	4.3	7.5	Alive
5	1	RSV	1	246	67	4.5	1.5	Alive
6	1	RSV	1	208	52	8.1	7.6	Alive
7	2	RSV	1	165	65	7.2	3.5	Alive
8	2	RSV	2:Cardiac Arrest, PNB 32 week	158	140	7.0	19.8	Alive
9	3	RSV	2, PNB 34 week	172	86	8.5	0.6	Alive
10	4	RSV	1	205	91	6.9	5.0	Alive
11	4	RSV	1	270	62	4.9	0.6	Alive
12	5	RSV + Pertussis	1	206	48	7.0	11.9	Alive
13	6	RSV	1	125	71	14.1	17.5	Alive
14	10	RSV	2: PNB 30 week	161	72	8.5	10.1	Alive
15	10	RSV	2:PNB 32 week HMD	80	60	24.9	6.6	Alive
16	11	(−)^a^	1	133	44	11.1	7.6	Alive

*Abbreviations*: *mo* months old, *PF ratio* PaO_2_/FIO_2_ ratio, *OI* oxygenation index, *PIM*_*2*_ Pediatric index of mortality 2, *RSV* respiratory syncytial virus, *PNB* preterm newborn, *HMD* hyaline membrane disease

^a^Viral Studies were negative, but pneumococcal superinfection was diagnosed

There were no complications related to the protocol. Table 2 shows ventilatory parameters and respiratory system mechanics for the study population: VTE 7.9 ± 1.4 mL·kg^−1^ ideal body weight, PEEP was 7.5 cmH2O (IQR 7–8.8), tPEEP 9 cmH2O (IQR 8–11), PIP 29 cmH2O (IQR 26–31), P_PL_ 24 cmH2O (IQR 20–26), IT 0.7 s (IQR 0.65–0.76), RR 28 bpm (IQR 26–30) and C_RS_ was 0.55 mL·cmH_2_O^−1^·kg^−1^ (IQR 0.44–0.89).

**Table 2:** Ventilatory parameters and respiratory system mechanics of infants with severe bronchiolitis

Number	FIO_2_	V_T_	RR	IT	PIP	P_PL_	PEEP	C_RS_	Raw_I_	Raw_E_
1	0.5	7.6	30	0.8	28	24	8	0.64	66	40
2	0.4	7.5	30	0.7	21	19.5	8	0.91	29	58
3	1	7	25	0.7	28	25	7	0.61	22	20
4	0.5	6.9	22	0.7	18	15	7	0.76	30	40
5	0.45	7.6	30	0.5	24	19	7	1.42	50	18
6	0.35	9.9	30	0.5	22	18	7	1.07	40	15
7	0.55	9.9	27	0.65	24	19	7	0.91	43	23
8	0.5	5.7	30	0.7	29	25	7	0.38	57	85
9	0.4	8,4	28	0.65	31	26	7	0.21	37	26
10	0.4	7.1	25	0.7	26	23	8	0.23	36	65
11	0.3	6,2	28	0.7	26	22	9	0.28	48	52
12	0.3	8	26	0.7	24	22	7	0.33	15	50
13	0.55	7.8	30	0.78	33	30	8	0.36	36	57
14	0.6	8.7	30	0.6	23	18	10	1.24	30	18
15	1	5.7	28	0.9	31	27	12	0.44	53	55
16	0.5	7.9	27	0.8	27	23	8	0.59	52	41
Median	0.5	7.6	28	0.7	29	24	7.5	0.55	38.5	40.5
IQR	0.15	0.95	3.25	0.07	4.5	6	1	0.56	20.5	33.25

*FiO*_*2*_ Fraction of inspired oxygen, %; *V*_*T*_ Tidal volume, ml·kg^−1^, *RR* respiratory rate, *IT* inspiratory time, seconds, *PIP* Peak inspiratory pressure, cmH2O, *PPL* Plateau pressure, cmH2O, *PEEP* Positive end-expiratory pressure, cmH2O, *C*_*RS*_ respiratory system static compliance, mL·cmH_2_O^−1^·kg^−1^, *Raw*_*I*_ inspiratory airway resistance, cmH_2_O·L^−1^·s^−1^, *Raw*_*E*_ expiratory airway resistance, cmH_2_O·L^−1^·s^−1^

Measurements of working pressure of the respiratory system parts were autoPEEP 1.5 (IQR 1–4.8) cmH_2_O, PIP–P_PL_ 5 cmH2O (IQR 0-11) and P_PL_ – autoPEEP 22.5 (IQR 15.2–25). This accounts of resistive 21.5% (CI95% 18.4–26.8), elastic 72.7% (CI95% 62.4–77.4%) and threshold 4.7% (CI95% 0–10.9) of total working pressure respectively. Elastic component of working pressure was significantly higher than resistive and both higher than threshold (*P* < 0.01).

Comparison between inspiratory and expiratory parameters showed that QI was significantly lower than QE [5 (4.27–6.75) L·min^−1^ v/s 16.5 (12–23.8) L·min^−1^, *P* < 0.05], but no significant differences were found between K_TI_ and K_TE_ [0.18 (0.12–0.30) v/s 0.18 (0.13–0.22) s]. However, RawI and RawE were not statistically different [38.8 (32–53) v/s 40.5 (22–55) cmH_2_O·L^−1^·s^−1^]. RawI was higher than RawE in 37.5% of cases and K_TI_:K_TE_ ratio was 1:1.04 (1:0.59–1.42).

There was a significant inverse relation between Raw_E_ and C_RS_ (*r* = 0.71, *P* = 0.001) (Additional file 2: Figure S1), but not with tPEEP (*r* = 0.36, *P* = 0.12), mean airway pressure (*r* = 0.45, *P* = 0.5) and Raw_I_ (*r* = 0.17, *P* = 0.48).

## Discussion

In this study, we measured pulmonary mechanics at bedside in infants requiring MV due to severe bronchiolitis, using the measurements provided by two conventional contemporary mechanical ventilators. Data obtained from these measurements show that the elastic component of the respiratory system (meaning the distal lung units and chest wall) and not the airway resistance is the main determinant of the work imposed upon MV. We calculated that almost three fourth parts of the total workload is generated to overcome the elastic component of the respiratory system. In addition, we found that the calculated resistance and time constant were similar during the expiratory and inspiratory phase, showing that an increase in expiratory resistance is not the main alteration in patients with severe bronchiolitis under mechanical ventilation.

These findings may seem unexpected and contradictory with the current understanding of severe bronchiolitis as a primarily obstructive airway disease with an increase in expiratory resistance, but they are supported by the observation done over a half century ago by Krieger et al. [13]. They observed a decrease in C_RS_ in spontaneous breathing infants with bronchiolitis when compared with healthy infants. More recent studies found the same alteration in C_RS_, most of the time attributed to significant air trapping and lung hyperinflation [14]. We were able to describe the threshold component in our patients, and autoPEEP was not clinically relevant, a finding comparable to that in previous studies [13, 14]. Threshold or autoPEEP is a minor contributor to the total working pressure [15]. When compared to the few reports of C_RS_ of infants without significant respiratory disease, patients of our study had consistently a decreased of C_RS_ [16].

Conflicting data exist with regard to the resistive airway component during severe bronchiolitis. Krieger et al. found that there were not clinical significant differences between inspiration and expiration in infants during bronchiolitis; even more, the latter was shortened. In that study measured K_TI_:K_TE_ ratio in children with bronchiolitis was higher than normal subjects (mean 1:1.1). They stated that this finding could be explained by Otis’ theory of the equality of time constants (which are the product of compliance and resistance) in a system with different airway’s caliber (unequally obstructed due to varying values of resistance), and flow is rapid in such a system, measured value of resistance would be the one of the larger airways [13]. It is important to realize that respiratory system mechanics measurements in patients under controlled positive pressure MV can be altered by the way the parameters are set. For instance, high ventilatory settings can elevate PIP, without P_PL_ changes [15]. A clinical study in 82 pediatric patients with bronchiolitis showed PIP oscillations between 25 and 45 cmH_2_O when children were ventilated with moderate to high V_T_ (10–15 ml·kg^−1^) [17]. An augmented V_T_ can also require longer expiratory times to allow the passive expiration, increasing the risk of air trapping and decreasing compliance due to hyperinflation. One of the strengths of our study is that MV settings were standardized and compatible with current standard of care: V_T_ and P_PL_ were limited and low Q_I_ were applied. This could have caused the low resistance to expiratory flow observed in our cohort. Normal values of Raw_E_ for this specific age group are scarce, but our patients’ Raw_E_ was lower than data from premature newborns without significant respiratory disease on spontaneous breathing [18]. Another consideration is the instrumentation used to measure the resistance in our study. When it is measured in a Y–piece, the resistive component of instrumental airway is included. De la Cruz et al. described in 21 patients that under these conditions the peak inspiratory pressure needed to ventilate the infant’s lungs is overestimated compared with actual airway pressure. It would be difficult to correct this overestimation since measurements with a tracheal catheter would be really challenging (or even contraindicated) in small infants [19].

An interesting finding was the inverse correlation between C_RS_ and Raw_E_. As previously reported in patients with ARDS, this observation shows that lungs with lower C_RS_ had higher elastic recoil [20]. This finding is concordant with our results, showing that during severe bronchiolitis the elastic component of working pressure is predominant, similar to ARDS pathophysiology. Hammer et al. described that ten out of 37 patients with severe bronchiolitis fulfilled the AECC criteria for ARDS [21, 22]. It is important to note that these infants had consolidation or infiltrates on 4 quadrants on the chest x-ray with a Murray’s lung injury score greater than 2.7. In our view, this group of RSV-induced ARDS is different from the cases we are describing. We excluded patients with more than 2 quadrants of infiltrates on chest x-ray (or obvious x-ray patterns of ARDS), and also all the measurements were done within 1 h after intubation. Hammer et al., don’t describe ventilatory parameters and timing of measurements. Because it was done more than 20 years ago, pre-low V_T_ era and contemporary care of acute respiratory failure, it is difficult to compare both case series. Surprisingly the duration of MV was greater than 14 days, compared to ours that was close to 5 days. We have to acknowledge that current PALICC consensus [23] definition includes a wide group conditions and pathologies under the brand ARDS, so some of our cases might be in the gray area of that ARDS definition, even when chest x-ray was not compatible with ARDS. On the other hand, bronchiolitis is a very heterogeneous condition including a wide spectrum of diseases. In our study, we aimed to describe a relatively homogenous group of ventilated infants with severe bronchiolitis with ad-hoc contemporary care.

It is important to emphasize that most bronchiolitis, even the most severe forms, do not require invasive mechanical ventilation with the contemporary care [24]. In our centers about 2% of bronchiolitis are admitted to a critical care unit, and between 30% and 50% of them require finally invasive mechanical ventilation. Our study is focus on that group of patients, infants with severe bronchiolitis requiring mechanical ventilation, so our pathophysiological findings may differ from moderate or mild disease.

Our study has some limitations. Our infants had a single disorder and the age range was large, thus our findings cannot be generalized to infants or children who have other disorders such as asthma, chronic lung disease or congenital heart disease. Reference values for healthy children in this age group has not been specifically reported and we did not include a control group, so comparisons and increments of the different components are only estimates. As previously commented, due to small size of patients we did not measure pleural pressure, so we could not determine the contribution of the chest to C_RS_. A single set of measurements, very close to intubation, was done, because we did not want to use long term or multiple doses of neuromuscular blockade. Also, patients were on controlled MV without respiratory muscle activity, can influence in the low Inspiratory resistance and measured autoPEEP, because patient efforts may increase the lung volume, facilitating hyperinflation [25], being frequent the coexistence between intrinsic PEEP and active expiration. Finally, we have to acknowledge that setting of MV parameters can directly modify the component of the equation of motion (i.e. Q_I_, RR, I:E ratio), but we tried to standardize the ventilatory setting during measurements.

Despite these limitations, we consider our findings in infants under mechanical ventilation with severe bronchiolitis are important in terms of the pathophysiological approach to this condition. Our analysis shows that the elastic component of the working pressure predominates rather than the airways resistance component. Future epidemiologic multicentric studies should address the different components of working pressure in severe bronchiolitis. A better understanding of respiratory system mechanics during mechanical ventilation may lead to change the traditional pharmacological and ventilatory approach to severe bronchiolitis as a predominant airway resistance disease.

## Conclusions

Analysis of respiratory mechanics measurements of infants with severe bronchiolitis receiving controlled MV shows that the preponderant constituent of the working pressure of the respiratory system is the elastic component. The elastic and resistive components in conjunction with flow profile are characteristic of restrictive diseases. A better understanding of the pathophysiology may lead to improve the traditional pharmacological and ventilatory approach of severe bronchiolitis as a predominant airway resistance disease.

## Additional files

Additional file 1: Table S1. Formulas for estimation of lung mechanics in quasi - static conditions. (DOCX 16 kb)

Additional file 2: Figure S1. Correlation between respiratory system compliance and expiratory airway resistance measured in children on mechanical ventilation due to severe bronchiolitis. (Abbreviations: C_RS_: respiratory system compliance; Raw_E_: expiratory airway resistance.) (TIFF 19195 kb)

## Abbreviations

∆P: Driving pressure
ARDS: Acute respiratory distress syndrome
C_RS_: Compliance respiratory system
K_TE_: Expiratory time constant
K_TI_: Inspiratory time constant
MV: Mechanical ventilation
Paw: Airway pressure
PEEP: Positive pressure at the end of exhalation
PIP: Peak inspiratory pressure
P_PL_: Plateau pressure
Q_E_: Maximum expiratory flow
Q_I_: Maximum inspiratory flow
Raw_E_: Resistance expiratory airway
Raw_I_: Resistance inspiratory airway
RSV: Respiratory syncytial virus
tPEEP: Total PEEP
V_T_: Tidal volume
WOB: Work of breathing

## References

[1] Fernandes RM, Andrade MG, Constant C, Malveiro D, Magalhães M, Abreu D, Azevedo I, Sousa E, Salgado R, Bandeira T (2016). Acute viral bronchiolitis: physician perspectives on definition and clinically important outcomes. Pediatr Pulmonol.

[2] Meissner HC (2016). Viral bronchiolitis in children. N Engl J Med.

[3] Bertrand P, Sánchez I (2007). Bronquiolitis aguda. Enfoque clínico de las Enfermedades Respiratorias del niño.

[4] Ralston SL, Lieberthal AS, Meissner HC, Alverson BK, Baley JE, Gadomski AM, Johnson DW, Light MJ, Maraqa NF, Mendonca EA, Phelan KJ, Zorc JJ, Stanko-Lopp D, Brown MA, Nathanson I, Rosenblum E, Sayles S 3rd, Hernandez-Cancio S. Clinical practice guideline: the diagnosis, management, and prevention of bronchiolitis. Pediatrics 2014; 134:e1474-e1502. 10.1542/peds.2014-2742 25349312

[5] Øymar K, Skjerven HO, Mikalsen IB (2014). Acute bronchiolitis in infants, a review. Scand J Trauma Resusc Emerg Med.

[6] Hasegawa K, Tsugawa Y, Brown DF, Mansbach JM, Camargo CA (2013). Trends in bronchiolitis hospitalizations in the United States, 2000-2009. Pediatrics.

[7] Leclerc F, Scalfaro P, Noizet O, Thumerelle C, Dorkenoo A, Fourier C (2001). Mechanical ventilatory support in infants with respiratory syncytial virus infection. Pediatr Crit Care Med.

[8] Rakshi K, Couriel JM (1994). Management of acute bronchiolitis. Arch Dis Child.

[9] Greenough A (2009). Role of ventilation in RSV disease: CPAP, ventilation, HFO, ECMO. Paediatr Respir Rev.

[10] Hess DR (2014). Respiratory mechanics in mechanically ventilated patients. Respir Care.

[11] Brochard L, Martin GS, Blanch L, Pelosi P, Belda FJ, Jubran A, Gattinoni L, Mancebo J, Ranieri VM, Richard JC, Gommers D, Vieillard-Baron A, Pesenti A, Jaber S, Stenqvist O, Vincent JL (2012). Clinical review: respiratory monitoring in the ICU - a consensus of 16. Crit Care.

[12] Cabello B, Mancebo J. Work of breathing. In: Hedenstierna G, Mancebo J, Brochard L, Pinsky M, (eds). Applied physiology in intensive care medicine. Berlin: Springer; 2009.

[13] Krieger I (1964). Mechanics of respiration in bronchiolitis. Pediatrics.

[14] Smith DW, Rector DM, Derish MT, Frankel LR, Ariagno RL (1990). Pulmonary function testing in infants with respiratory syncytial virus bronchiolitis requiring mechanical ventilation. Pediatr Infect Dis J.

[15] Smith PG, el-Khatib MF, Carlo WA (1993). PEEP does not improve pulmonary mechanics in infants with bronchiolitis. Am Rev Respir Dis.

[16] Tepper RS, Pagtakhan RD, Taussig LM (1984). Noninvasive determination of total respiratory system compliance in infants by the weighted-spirometer method. Am Rev Respir Dis.

[17] Frankel LR, Lewiston NJ, Smith DW, Stevenson DK (1986). Clinical observations on mechanical ventilation for respiratory failure in bronchiolitis. Pediatr Pulmonol.

[18] Gerhardt T, Reifenberg L, Duara S, Bancalari E (1989). Comparison of dynamic and static measurements of respiratory mechanics in infants. J Pediatr.

[19] De la Cruz RH, Banner MJ, Weldon BC (2005). Intratracheal pressure: a more accurate reflection of pulmonary airway pressure in pediatric patients with respiratory failure. Pediatr Crit Care Med.

[20] Pesenti A, Pelosi P, Rossi N, Virtuani A, Brazzi L, Rossi A (1991). The effects of positive end-expiratory pressure on respiratory resistance in patients with the adult respiratory distress syndrome and in normal anesthetized subjects. Am Rev Respir Dis.

[21] Hammer J, Numa A, Newth CJ (1997). Acute respiratory distress syndrome caused by respiratory syncytial virus. Pediatr Pulmonol.

[22] Bernard GR, Artigas A, Brigham KL, Carlet J, Falke K, Hudson L, Lamy M, Legall JR, Morris A, Spragg R, and the Consensus Committee (1994). The American-European consensus conference on ARDS. Definition, mechanisms, relevant outcomes, and clinical trial coordination. Am J Respir Crit Care Med.

[23] Pediatric Acute Lung Injury Consensus Conference Group (2015). Pediatric acute respiratory distress syndrome: consensus recommendations from the pediatric acute lung injury consensus conference. Pediatr Crit Care Med.

[24] Milési C, Essouri S, Pouyau R, Liet JM, Afanetti M, Portefaix A, Baleine J, Durand S, Combes C, Douillard A, Cambonie G, Groupe Francophone de Réanimation et d’Urgences Pédiatriques (GFRUP) (2017). High flow nasal cannula (HFNC) versus nasal continuous positive airway pressure (nCPAP) for the initial respiratory management of acute viral bronchiolitis in young infants: a multicenter randomized controlled trial (TRAMONTANE study). Intensive Care Med.

[25] Larouche A, Massicotte E, Constantin G, Ducharme-Crevier L, Essouri S, Sinderby C, Beck J, Emeriaud G (2015). Tonic diaphragmatic activity in critically ill children with and without ventilatory support. Pediatr Pulmonol.

