# Supplementary material for: Respiratory mechanics in infants with severe bronchiolitis on controlled mechanical ventilation
Source: BMC Pulm Med. 2017 Oct 6;17:129. doi: 10.1186/s12890-017-0475-6 (PMC6389183; doi:10.1186/s12890-017-0475-6)
Supplement: Supplementary file 1 — Formulas for estimation of lung mechanics in quasi - static conditions. (DOCX 16 kb) [file 12890_2017_475_MOESM1_ESM.docx]

Additional file 1 Table S1. Equations and calculations for lung mechanics in quasi - static conditions.

| AutoPEEP: Intrinsic PEEP  C_RS_: Compliance respiratory system  ΔP: Driving Pressure  Paw: Airway Pressure  PEEP: (set) Positive pressure at the end of exhalation  PIP: Peak inspiratory pressure  P_PL_: Plateau pressure  QI: Maximum inspiratory flow  QE: Maximum expiratory flow  Raw_E_: Resistance expiratory airway  RawI: Resistance inspiratory airway  K_TI_: Inspiratory time constant  K_TE_: Expiratory time constant  tPEEP: Total PEEP  V_T_: Tidal volume | **Equation of Motion:**  Paw = V_T_ / CRS + RawI · QI + autoPEEP  **Resistive component**  RawI = (PIP - PPL) / QI  Raw_E_ = (PPL - tPEEP) / QE  **Elastic component:**  ΔP = PPL - tPEEP  CRS = V_T_ / ΔP  **Threshold Component:**  autoPEEP = tPEEP - PEEP  **Time constants:**  K_TI_ = CRS · RawI  K_TE_ = CRS · RawE |
| --- | --- |
