# Supplementary figures and images for: Respiratory mechanics in infants with severe bronchiolitis on controlled mechanical ventilation
Source: BMC Pulm Med. 2017 Oct 6;17:129. doi: 10.1186/s12890-017-0475-6 (PMC6389183; doi:10.1186/s12890-017-0475-6)

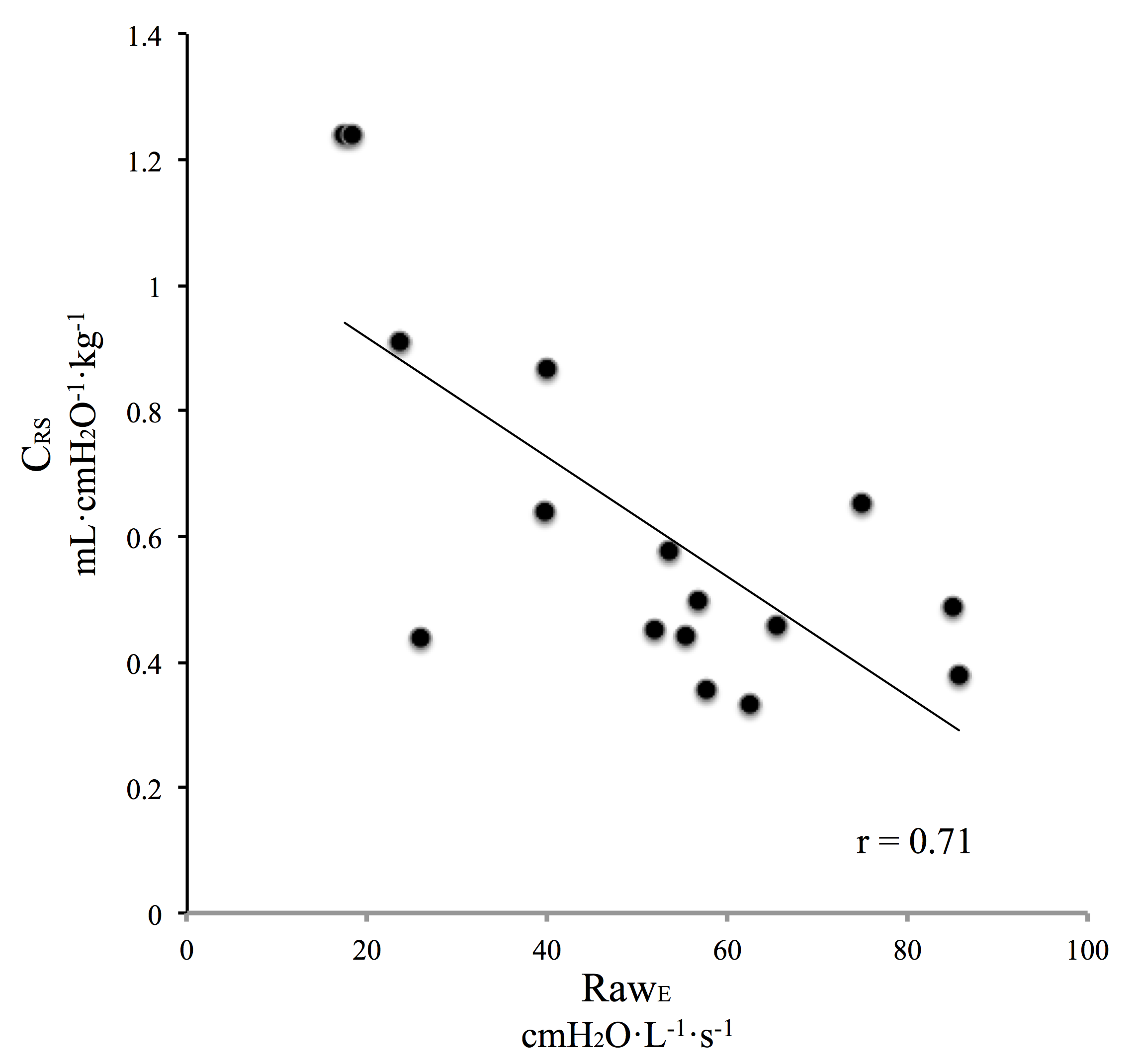

Supplement: Supplementary file 2 — Correlation between respiratory system compliance and expiratory airway resistance measured in children on mechanical ventilation due to severe bronchiolitis. (Abbreviations: CRS: respiratory system compliance; RawE: expiratory airway resistance.) (TIFF 19195 kb) [file 12890_2017_475_MOESM2_ESM.tiff]
